# Value of peripheral blood count for dengue severity prediction

**DOI:** 10.1186/s13104-018-3505-4

**Published:** 2018-06-20

**Authors:** Udaya Ralapanawa, A. T. M. Alawattegama, Malinga Gunrathne, Sampath Tennakoon, S. A. M. Kularatne, Thilak Jayalath

**Affiliations:** 1 Department of Medicine, Faculty of Medicine, University of Peradeniya, Peradeniya, Sri Lanka; 2 Department of Community Medicine, Faculty of Medicine, University of Peradeniya, Peradeniya, Sri Lanka

**Keywords:** Dengue fever, Dengue haemorrhagic fever, Peripheral blood parameters, Acute febrile phase, Critical phase

## Abstract

**Objective:**

This retrospective study was conducted in 2017 with the objective of evaluating the value of acute phase peripheral blood parameters in predicting dengue haemorrhagic fever (DHF). Patients, who were admitted to Teaching Hospital Peradeniya between January and August 2017 due to dengue illness, were recruited into this study.

**Results:**

A total of 515 patients participated in the study. Among them, 333 were DHF patients while 182 patients were managed as DF. There was a significant difference in mean values of platelets and haemoglobin observed during acute phase in non-leakers compared to the patients who progressed to DHF, while no significant difference was observed for white blood cells, neutrophils, lymphocytes and haematocrit values. A significantly higher mean value was observed in white blood cells and hemoglobin in leakers compared to non-leakers during day 5. Mean day 5 platelet value was significantly lower among leakers compared to non-leakers but no significant difference between haematocrit, neutrophil and lymphocyte values were observed. ROC curve performed for acute phase platelet values and haemoglobin values to gain a predictive value for female and male DHF patients and cut off values with high sensitivity and specificity to predict DHF could be obtained for the platelet count.

**Electronic supplementary material:**

The online version of this article (10.1186/s13104-018-3505-4) contains supplementary material, which is available to authorized users.

## Introduction

Dengue fever (DF) is a mosquito-borne tropical disease caused by the Dengue virus [1–4]. Dengue is currently a major public-health concern throughout tropical and sub-tropical regions of the world including Sri Lanka [1, 5]. It has made a devastating impact on public health in Sri Lanka for several decades [6].

According to the Epidemiology unit, Ministry of health, Sri Lanka, 165,517 suspected dengue cases have been reported from January to October 2017, which is more than twice the number in 2016 where 55,150 cases were reported [7]. A total number of 403 dengue deaths have been reported from January to October 2017 [7]. There is a decline of dengue deaths over the last two decades despite the rising case incidence [6, 8], which indicates that the in-ward management practice has improved over the time. However the change of predominant circulating dengue serotype in population, causing changes of clinical pattern of dengue [8] and the rising case incidence highlights the need for more knowledge and resources for both preventive and curative aspects of the disease.

Dengue has a wide spectrum of clinical presentations, often with unpredictable clinical evolution and outcome. While most patients recover following a self-limiting non-severe clinical course, a small proportion progress to severe disease, mostly characterized by plasma leakage with or without haemorrhage [9, 10].

Peripheral blood parameters change during the course of the illness. Dengue fever is characterized by leucopenia (White Blood Cells (WBC) < 5000 cells/mm^3^), thrombocytopenia (< 150,000 cells/mm^3^), rising haematocrit (5–10%) and there should be no evidence of plasma leakage [10].

In Dengue Hemorrhagic Fever (DHF) there is evidence of plasma leakage which is usually evidenced by ascites or pleural effusion. Peripheral blood parameters characterized by thrombocytopenia (< 100,000 cells/mm^3^) and haematocrit rise > 20%. A drop in platelet count below 100,000 cells/mm^3^, may be occasionally observed in dengue fever but is almost a feature in DHF [7]. There is also a tendency towards haemorrhage associated with severe thrombocytopenia [10]. Dengue haemorrhagic fever can be further classified into four grades [10].

Clinical course of dengue haemorrhagic fever consists of three stages [9–11],

- Febrile phase (lasting 2–7 days)
- Critical phase (Leaking phase) (24–48 h)
- Convalescent phase (2–4 days)

Early identification of individuals likely to progress to severe disease is difficult. Therefore large numbers of patients with possible dengue get admitted to hospitals, primarily for observation, exceeding the limited resources at the government hospitals, whereas only a small proportion of patients only need specialized care [12].

### Objectives

In this study our objective was to predict dengue hemorrhagic fever using peripheral blood parameters of the acute febrile phase of the illness.

## Main text

### Method

Ethical clearance was obtained from the institutional ethical review committee. During dengue fever epidemic this year, nearly about half of the dengue patients in Kandy district were managed at the Teaching Hospital Peradeniya which is a tertiary care clinical setting. From January to August 2017, 515 patients who got admitted to Teaching Hospital Peradeniya with positive serology for dengue (Positive NS1 or positive IgM and IgG) were recruited to this study after obtaining informed written consent. Only the patients admitted to the ward in the acute febrile phase of the illness were recruited. They were retrospectively reviewed for demographic, serial clinical, laboratory and outcome data.

Cases were classified mainly into DF and DHF, and DHF group further classified into four grades. Other conditions that can cause changes in full blood count such as haematological and reticular endothelial disorders and those who were on cytotoxic drugs were excluded from the study. Furthermore patients who developed complications of dengue infection like acute liver failure, myocarditis, bleeding were also excluded from the analysis. All of the patients were managed according to the national guidelines on management of dengue fever and DHF in Sri Lanka [9].

Data was collected retrospectively from bed head tickets using routine blood investigation reports which were carried out during their hospital stay in acute phase (day 2, 3) and day 5 of illness. A data sheet was used to collect data for the variables of full blood count parameters.

### Statistical analysis

Data was analyzed using IBM-SPSS version 16 statistical package. Monovariate analysis was used to describe the study sample. Students t test was used to explore differences between mean peripheral blood parameters within the acute phase and the critical phase. ROC curves were used to look for cut off values for possible predictors based on bivariate analysis. Linear regression was used to explore the trends in the parameters over the course of the disease.

### Results

Approximately 515 patients data were used in the study. Of them 343 were males and 172 were females. Mean age of the population was 32.8 with a standard deviation of 14.4. Ages of the patients ranged from 14 to 86 years.

Our study population consisted of 182 patients who did not develop DHF during their hospital stay and 333 patients who progressed to DHF in the ward.

During the acute febrile phase (Day 2, 3) of the illness, leucopenia (WBC < 5000 cells/mm^3^) was observed among 70.9% of patients. When considering the values of acute febrile phase, average WBC values for DF patients and those who eventually progressed to DHF were 4.38 and 4.49, respectively (Table 1). Neutrophils, a component of WBC showed mean values of 2.90 and 3.17 among DF and DHF patients, respectively. Mean lymphocyte counts for DF and DHF were 1.03 and 0.89, respectively (Table 1).

**Table 1:** Distribution FBC during the acute febrile phase (day 2 and 3 of illness)

	DF/DHF	No.	Mean	Standard deviation	P value
WBC (cells/mm^3^)	DF	182	4.38	2.2	0.62
	DHF	333	4.49	2.29	
Neutrophil (cells/mm^3^)	DF	182	2.90	1.88	0.13
	DHF	332	3.17	1.99	
Lymphocyte (cells/mm^3^)	DF	55	1.03	0.6	0.20
	DHF	279	0.89	0.79	
PCV (%)	DF	182	42.9	32.18	0.63
	DHF	333	44.50	37.66	
Platelet (cells/mm^3^)	DF	182	154.36	55.03	< 0.001
	DHF	332	115.59	64.3	
Haemoglobin (g/dL)	DF	180	13.61	1.52	0.02
	DHF	324	14.07	2.43	

During acute febrile phase haemoglobin levels ranged from 9.5 to 18.8 mg/dL with average values for DF and DHF being 13.61 and 14.07, respectively. Mean packed cell volume (PCV) values for DF and DHF were 42.90 and 44.50, respectively. Thrombocytopenia (platelet < 150,000 cells/mm^3^) was observed in 63.1% of patients during acute febrile phase and 33.2% had platelet counts below 10,0000 cells/mm^3^. Mean platelet values for DF and DHF were 154.36 and 115.59, respectively (Table 1).

The blood samples of the 5th day of the illness were analyzed similarly for the peripheral blood parameters. When considering the 5th day values, mean WBC values among leakers and non-leakers were 3.30 and 4.05, respectively. Mean neutrophil values for DF and DHF were 1.88 and 2.12, respectively. Mean lymphocyte counts for DF and DHF were 1.70 and 2.48, respectively (Table 2).

**Table 2:** Distribution of FBC on day 5 of illness

	DF/DHF	No.	Mean	Standard deviation	P value
WBC (cells/mm^3^)	DF	181	3.30	1.82	0.001
	DHF	333	4.04	2.58	
Neutrophil (cells/mm^3^)	DF	182	1.88	4.77	0.52
	DHF	333	2.12	3.62	
Lymphocyte (cells/mm^3^)	DF	55	1.69	3.3	0.60
	DHF	278	2.48	11.01	
PCV (%)	DF	182	39.29	23.32	0.37
	DHF	333	41.07	20.13	
Platelet (cells/mm^3^)	DF	181	108.5	53.6	< 0.001
	DHF	331	47.49	41.22	
Haemoglobin (g/dL)	DF	180	13.12	1.46	< 0.001
	DHF	328	14.04	1.84	

Haemoglobin values ranged from 9.00 to 18.20 during the 5th day of illness with average values of 13.12 and 14.04 among DF and DHF patients, respectively. Mean PCV values for DF and DHF were 39.29 and 41.07, respectively. Mean platelet counts for DF and DHF were 108.50 and 47.49, respectively (Table 2).

Analytical results showed that the leakers had significantly different mean values of haemoglobin and platelet counts during acute phase compared to non-leakers. There was no significant difference between acute phase WBC, neutrophils, lymphocytes and PCV values between DF and DHF patients.

WBC and haemoglobin values were significantly higher among leakers compared to non-leakers during day 5 and platelet values were significantly lower among leakers. But no significant difference between day 5 neutrophils, lymphocyte and PCV values were observed.

Summary of blood parameters of DF and DHF patients according to gender was summarized in Table 3. When considering the assessed parameters by sex, differences were similarly distributed as per the total population except WBC among males which was higher among DHF patients on 5th day and Hemoglobin which was not significantly different among females on the 3rd day (Table 3).

**Table 3:** Distribution of FBC parameters during the acute febrile phase (Day 2 and 3 of illness) and Day 5 of illness according to gender

	Acute febrile phase (day 2 and 3) of illness					Day 5 of illness				Reference ranges for blood parameters
	DF/DHF	No.	Mean	Standard deviation	P value	No.	Mean	Standard deviation	P value	
Male										
WBC (cells/mm^3^)	DF	126	4.4	2.0	0.40	126	3.4	1.9	0.01	4.3–10*10^3^/μL
	DHF	216	4.6	2.1		216	3.9	2.2		
Neutrophil (cells/mm^3^)	DF	126	2.7	1.7	0.01	126	2.1	5.6	0.75	1.8–7.0*10^3^/μL
	DHF	215	3.3	2.0		216	1.9	1.0		
Lymphocyte (cells/mm^3^)	DF	43	1.0	0.61	0.19	43	1.8	3.7	0.77	1.0–4.8*10^3^/μL
	DHF	180	0.91	0.8		180	2.1	4.9		
PCV (%)	DF	126	44.1	34.3	0.34	126	41.5	27.3	0.79	38–50%
	DHF	216	48.2	46.0		216	42.2	6.6		
Platelet (cells/mm^3^)	DF	126	146.3	46.6	< 0.01	126	104.8	51.7	< 0.01	150–400*10^3^/μL
	DHF	215	113.1	63.4		214	46.7	45.1		
Haemoglobin (g/dL)	DF	125	14.0	1.3	< 0.01	126	13.6	1.2	< 0.01	13–18*10^6^/μL
	DHF	210	14.8	2.5		213	14.8	1.5		
Female										
WBC (cells/mm^3^)	DF	54	4.2	2.2	0.82	54	3.0	1.5	0.002	4.3–10*10^3^/μL
	DHF	117	4.1	2.3		117	4.1	3.1		
Neutrophil (cells/mm^3^)	DF	54	3.1	2.0	0.55	54	1.3	0.7	0.05	1.8–7.0*10^3^/μL
	DHF	117	2.9	1.8		117	2.4	5.9		
Lymphocyte (cells/mm^3^)	DF	11	1.0	0.5	0.41	11	1.0	0.6	0.21	1.0–4.8*10^3^/μL
	DHF	99	1.0	0.6		98	3.2	17.3		
PCV (%)	DF	54	39.9	26.9	0.52	54	33.7	5.7	0.09	36–45%
	DHF	117	37.5	7.1		117	39.0	32.7		
Platelet (cells/mm^3^)	DF	54	172.8	68.2	< 0.01	54	116.6	57.6	< 0.001	150–400*10^3^/μL
	DHF	117	120.2	65.9		117	48.8	33.1		
Haemoglobin (g/dL)	DF	54	12.5	1.4	0.62	53	11.8	1.2	< 0.001	11.5–15.5*10^6^/uL
	DHF	114	12.7	1.5		115	12.6	1.3		

ROC curve was performed separately for female and male patients for acute phase platelet values and haemoglobin values to gain a predictive value for DHF during the acute febrile phase. ROC curve performed for female patients shows area under the curve was 0.71 for platelets and 0.50 for haemoglobin. A cut off value with high sensitivity and specificity to predict DHF could be obtained from our sample platelets (see Additional file 1: Figure S1; https://figshare.com/s/beec71ea2bef983d335b). We propose a cut off of 116,000 as a reasonable cut off for women to predict progression into DHF from DF at a sensitivity of 83% and a specificity of 56%. Hemoglobin may not be used since the area under the curve is less than 0.7.

Similar result was observed by ROC curve performed for acute phase platelet values and haemoglobin values to gain a predictive value for DHF of male patients. Area under the curve was 0.70 for platelets and 0.40 for haemoglobin for male DF patients. We propose a cut off of less than 105,000 for platelets to predict progression into DHF with sensitivity of 80 and 50% specificity (see Additional file 2: Figure S2; https://figshare.com/s/beec71ea2bef983d335b).

### Discussion

Dengue fever has a highly variable disease evolution and outcome. The purpose of this study was to predict DHF, which may lead to severe outcome, using acute phase peripheral blood parameters. Because the research was conducted mainly during a major dengue outbreak in the country and also in a Teaching hospital a sample size of 515 was obtained with a number of 333 DHF patients.

Platelets contribute to increased vascular permeability by inflammation dependent release of IL-1β [13]. A rapid decrease in platelet count, concomitant with a rising haematocrit, is suggestive of progression to plasma leakage [12]. Even though previous studies have shown lower platelet values among DHF patients [14–17], only few study findings reflect acute febrile phase platelet values. In our study among DHF patients a significant drop of the mean platelets value was observed compared to non-leakers even during the acute febrile phase, suggesting a rapid decline of platelet counts before the critical phase. Similar to our findings, a previous research done in the paediatric population, acute febrile phase platelet counts in the patients who developed DSS tended to be lower than the patients who never progressed to DHF [12].

Heamo-concentration is a well-known finding of DHF [10, 11]. The significantly higher haemoglobin values observed among leakers during both acute phase and day 5 can be explained by the concentration of plasma due to fluid leakage, which causes a rise of haemoglobin weight in a unit volume of blood. When plasma leakage through the blood vessels occur haematocrit value is also expected to rise [10, 11, 14, 17], but a significant difference was not observed for PCV among dengue fever and DHF patients in our study. Furthermore the day 5 mean haematocrit values was lower than day 3 values in both leakers and non-leakers in contrast to the expectation, whereas a previous study showed a rising trend of mean haematocrit values until 5th day of illness [17]. The decline of the mean value may be due to the fluid management in the ward where given fluid quotas may have caused some dilution of the plasma. Similar to our findings a previous study done in paediatric population also found no clear difference in daily haematocrit levels between study participants who did and who did not develop DSS [12].

Leucopenia is usually observed in the course of dengue fever [7, 9–11, 17–19]. In our study both DF and DHF patients showed mean values less than 5000 cells/mm^3^. Even though acute phase WBC values and neutrophil values were arithmetically higher among DHF patients, they cannot be used to predict DHF in acute phase because no statistical significance was observed. A previous study suggested absence of leucopenia as a predictor of severe dengue [20] but this is in contrast to our findings where only significant difference was observed during day 5 values.

Lymphocytes mean values were within the normal range, though it was expected to rise during many viral infections [21, 22]. This is in contrast to a previous research finding where there was a significant increase in both the percentage and the concentration of total lymphocytes in dengue fever, which was due to a marked increase in both the percentage and the number of atypical lymphocytes, where normal lymphocytes were essentially unchanged [19].

### Conclusions

Our objective of this study was to predict dengue hemorrhagic fever using peripheral blood parameters of the acute febrile phase of the illness. Thrombocytopenia during acute febrile phase is a predictor of DHF. Patients with low platelets in acute febrile phase of dengue fever need more medical attention as there is a higher chance of progression to DHF. But WBC counts, Hb, Neutrophils, lymphocytes and PCV counts in acute febrile phase are unlikely to predict DHF. Also this provides strong support for the WHO recommendation to perform daily full blood counts in Dengue patients.

## Limitations

The current study was restricted to only hospitalized patients and conducted in a single hospital. If the patients those who got treated at the Out Patient Department level were also included, that could have caused some changes in the results because mainly the patients who fulfill the criteria for admission got admitted to the wards.

## Additional files

**Additional file 1: Figure S1.** Discriminatory function of day 3 platelet count and haemoglobin concentration in predicting progression into dengue haemorrhagic fever among female dengue patients. Area under the curve was 0.71 for platelets and 0.50 for haemoglobin. A cut off value with high sensitivity and specificity to predict DHF could be obtained from our sample.

**Additional file 2: Figure S2.** Discriminatory function of day 3 platelet count and haemoglobin concentration in predicting progression into dengue haemorrhagic fever among male dengue patients. Area under the curve was 0.70 for platelets and 0.40 for haemoglobin. A cut off value with high sensitivity and specificity to predict DHF could be obtained from our sample.

## Abbreviations

DF: dengue fever
WBC: white blood cells
DHF: dengue haemorrhagic fever
PCV: packed cell volume
