# Supplementary figures and images for: Value of peripheral blood count for dengue severity prediction
Source: BMC Res Notes. 2018 Jun 20;11:400. doi: 10.1186/s13104-018-3505-4 (PMC6011352; doi:10.1186/s13104-018-3505-4)

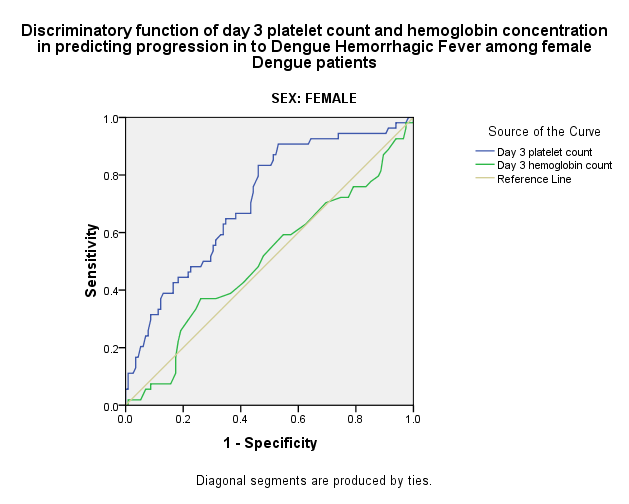

Supplement: Supplementary file 1 — Additional file 1: Figure S1. Discriminatory function of day 3 platelet count and haemoglobin concentration in predicting progression into dengue haemorrhagic fever among female dengue patients. Area under the curve was 0.71 for platelets and 0.50 for haemoglobin. A cut off value with high sensitivity and specificity to predict DHF could be obtained from our sample. [file 13104_2018_3505_MOESM1_ESM.docx]

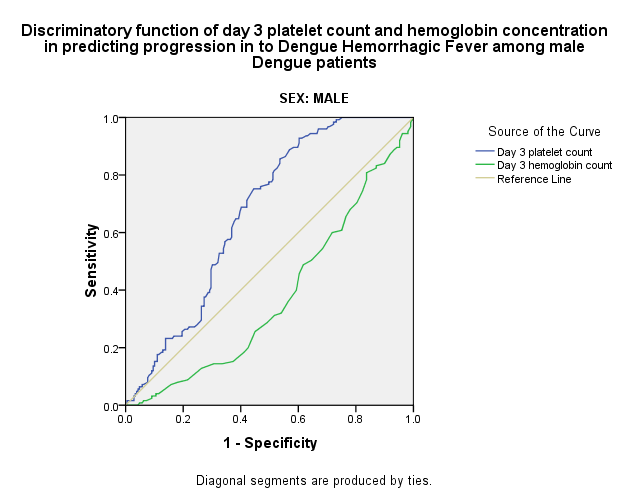

Supplement: Supplementary file 2 — Additional file 2: Figure S2. Discriminatory function of day 3 platelet count and haemoglobin concentration in predicting progression into dengue haemorrhagic fever among male dengue patients. Area under the curve was 0.70 for platelets and 0.40 for haemoglobin. A cut off value with high sensitivity and specificity to predict DHF could be obtained from our sample. [file 13104_2018_3505_MOESM2_ESM.docx]
